# Neutrophil extracellular traps as drivers of epithelial-mesenchymal transition in cancer cells

**DOI:** 10.3389/fimmu.2025.1655019

**Published:** 2025-10-09

**Authors:** Maurizio Maddalena, Jelena Dimitrov, Tayyaba Mehmood, Cristina Terlizzi, Paola Maria Helena Esposito, Antonella Franzese, Sara Pellegrino, Viviana De Rosa, Francesca Iommelli, Silvana Del Vecchio

**Affiliations:** ^1^ Department of Advanced Biomedical Sciences, University “Federico II”, Naples, Italy; ^2^ Institute of Biostructures and Bioimaging, National Research Council, Naples, Italy; ^3^ Azienda Ospedaliera Universitaria Federico II, Naples, Italy

**Keywords:** neutrophil extracellular traps, epithelial-mesenchymal transition, metastatic dissemination, signalling pathways, NETosis

## Abstract

Neutrophil extracellular traps (NETs) are complex structures released by activated neutrophils and composed by double-stranded DNA associated with histones and an arsenal of proteases and proteins. NETs are reported to be present in tumors and blood of cancer patients where they can directly or indirectly modulate different functions of cancer cells. Here, we will summarize the current evidences indicating that NETs can drive tumor growth and metastatic dissemination through different signaling pathways. Many studies reported that NETs can enhance cancer cell proliferation and promote colonization of distant sites by circulating cancer cells, especially in the presence of sepsis and surgical stress. However, there are scattered reports on the ability of NETs to induce epithelial-mesenchymal transition (EMT) in different contexts. In this minireview, we will focus especially on the studies investigating the induction of EMT by NETs trying to highlight the involvement of specific signaling pathways. The results of these studies delineate an intricate scenario in which NETs stay at the crossroad between inflammation and cancer playing a leading role in metastatic dissemination by inducing EMT through different signaling pathways.

## Introduction

Neutrophil extracellular traps (NETs) are filamentous structures released by activated neutrophils to entrap and kill pathogens (1). Beyond this primary function, it has been progressively clear that these extracellular particles have multiple functions in a number of pathological states including autoimmunity (2, 3), wound healing (4), thrombotic disease (5, 6); and cancer progression (7, 8). The multifaceted effects of NETs rely upon their complex structure that includes a backbone of double-stranded DNA associated with histones and an arsenal of proteases such as myeloperoxidase (MPO), neutrophil elastase (NE), matrix metalloproteinase-9 (MMP-9), cathepsin G (CG) and pentraxin 3 (PTX3). NETs are released not only in response to many different pathogens but also in sterile conditions in response to inflammatory mediators such as interleukin-8, CXCL1 and other chemokines (9), granulocyte colony-stimulating factor (G-CSF) (10) and cathepsin C (11).

**Figure 1:**
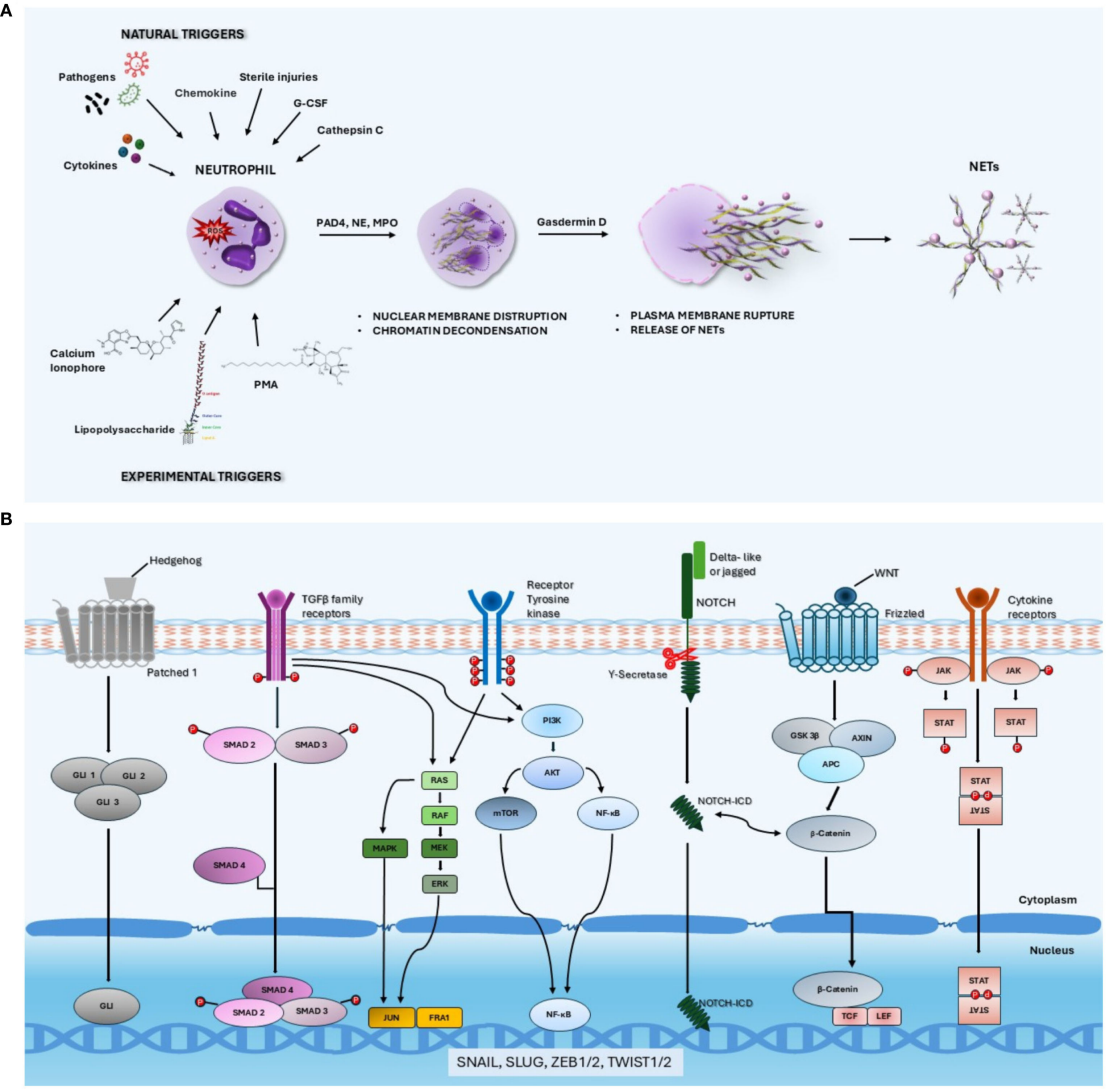
**(A)** Schematic representation of lytic NETosis. NET formation can be triggered by many natural agents including pathogens, cytokines/chemokines, granulocyte colony-stimulating factor and cathepsin C as well as by chemical compounds such as phorbol myristate acetate, calcium ionophores and lipopolysaccharide. The process of NET formation requires ROS production followed by PAD4-mediated histone citrullination and chromatin decondensation induced by NE and MPO. The subsequent steps are the disassembly of nuclear membrane and fusion of the nuclear content with the cytotoxic agents released from the cytoplasmatic granules followed by the disruption of the plasma membrane involving gasdermin-D polymerization. **(B)** Signaling pathways involved in the activation of epithelial-mesenchymal transition. The signaling pathways that are reported to activate the epithelial-mesenchymal transition program include WNT, Hedgehog, NOTCH, TGFβ, tyrosine kinase receptors and cytokine receptors downstream signaling cascades. The specific binding of several ligands to membrane receptors triggers intracellular signals converging on a pool of transcription factors including Zeb family members, Snail, Slug and Twist 1 that orchestrate a series of events ultimately resulting in loss of epithelial characteristics and acquisition of a mesenchymal phenotype.

Phorbol myristate acetate, calcium ionophores and lipopolysaccharyde are experimental inducers of NET formation (12). The process leading to NET formation requires ROS production triggered by the majority of NET stimulators, followed by peptidylarginine deiminase 4 (PAD4)-mediated histone citrullination and chromatin decondensation induced by activated neutrophil elastase (NE) and myeloperoxidase (MPO) (**Figure 1A**) (13). The subsequent step is the disruption of nuclear membrane and fusion of the nuclear content with the cytotoxic arsenal released from the cytoplasmatic granules. Following the disruption of the plasma membrane involving gasdermin-D polymerization (14), NETs are released into the extracellular space. The final step of this process usually implies the lytic death of neutrophil, termed netosis. However, in some circumstances, NETs can be generated by the extrusion of chromatin and granular proteins through vesicular transport without the production of ROS and disruption of plasma membrane. This process termed vital netosis leaves the neutrophil without nucleus but alive (15).

In the last decade, an increasing number of studies evaluated the role of NETs in human cancers based on the consideration that they may mediate the cross-talk between cancer cells and tumor microenvironment. Here we will summarize the evidences indicating their function in driving tumor growth and metastatic dissemination of cancer cells with a special focus on the ability of NETs to induce the epithelial-mesenchymal transition.

## NETs as drivers of tumor growth

NETs have been detected in the extracellular space of primary tumors and metastatic sites of human cancers as well as in the blood of cancer patients and they have been reported to contribute directly or indirectly to cancer growth and progression. Several observations indicate that NETs can affect cancer cell proliferation by protease ECM remodelling and consequent release of growth factors or activation of ligands for specific receptors (11, 16). For instance, NET-derived NE and MMP-9 degraded laminin in the ECM causing the exposure of an epitope that can bind to integrin α3β1 on dormant cancer cells with the consequent activation of downstream signaling pathways and induction of proliferation. Also, the DNA component was reported to enhance cancer cell proliferation by interacting with DNA receptors. Yang et al. reported that NETs can bind to cancer cells through CCDC25 receptor and activate the integrin linked kinase (ILK)-β-PARVIN-RAC1-CDC42 cascade in breast cancer cells thus enhancing motility, invasion and proliferation (17).

NETs induced a significant increase of the proliferation rate in diffuse large B-cell lymphoma cells through the activation of TLR9 receptor and downstream NFkB, STAT3 and p38 signaling pathways (18). These effects on proliferation were abrogated by NETs digestion with DNase I or pre-treatment of neutrophils with NE inhibitors. A dose-dependent enhancement of proliferation was observed in Panc02 murine pancreatic cancer cells exposed to NET supernatant (19). Furthermore, in a murine subcutaneous tumor model, NETs caused stellate pancreatic cells proliferation and protease secretion through activation of RAGE signaling (19).

An additional structural component of NETs is the protein High Mobility Group Box 1 (HMGB1). Glioblastoma cells exposed to NETs showed an enhanced proliferation rate due to the interaction of NET-derived HMGB1 with RAGE (20). Furthermore, in colorectal cancer cells, NET-derived HMGB1 was shown to interact with TLR9 with the subsequent activation of MAP kinase pathway (21).

In murine models, subcutaneous or intrasplenic injection of colorectal cancer cells caused a more rapid development of tumors and liver metastases in wild type animals than in PAD4-KO mice that are not able to induce NETosis (22). In the same study, the authors showed that these effects were due to the activation of TLR4 on cancer cells by NET-derived neutrophil elastase causing PGC1α upregulation and enhanced mitochondrial biogenesis.

## NETs as drivers of metastatic dissemination

NETs were initially thought to promote the development of distant metastases by mechanically entrapment of circulating tumor cells. In an animal model of sepsis, the deposition of NETs within hepatic sinusoidal spaces was associated with an increased number of hepatic micrometastases that evolved in gross metastatic lesions following the intrasplenic injection of cancer cells (23). Subsequent studies showed that the binding of cancer cells to NETs was not simply mechanical but was mediated by the interaction of NET-DNA to the CCDC25 receptor (17) or the binding of NET-associated fibronectin to beta 1 integrins expressed on the plasma membrane of cancer cells (24, 25).

Furthermore, in an ischemia and reperfusion murine model, surgical stress caused NET formation that in turn promoted the development of liver metastases of colorectal cancer cells through the binding of NET-derived HMGB1 to TLR9 and activation of MAP kinases pathway (21). Similarly, in a murine model of surgical stress, the systemic injection of colorectal cancer cells caused a 3-fold higher number of lung metastases than in the control group (26). In this study, surgical stress caused NETs formation and deposition in lung microvasculature as well as platelet activation with the formation of platelet-tumor cell aggregates that facilitated NET-mediated entrapment of cancer cells in lung tissue. Blocking platelet activation by inhibiting the ERK5-GPIIb/IIIa integrin-dependent pathway or knocking out TLR4 avoided lung metastases formation.

Additional studies showed that NETs promote migration and invasion of cancer cells thus facilitating both the intravasation and extravasation of cancer cells. Co-culturing breast cancer cells with neutrophils enhanced cell invasion *in vitro* and this effect was abrogated by treatment with DNase I or inhibitors of NET formation (27). In the same study, treatment with nanoparticles coated with DNase I strongly reduced the formation of lung metastases in mice.

## NETs as drivers of epithelial-mesenchymal transition

The epithelial-mesenchymal transition (EMT) is a reversible biological process in which epithelial cells lose their peculiar characteristics and acquire a mesenchymal phenotype. It is not a binary process but a progressive transition through all possible states between epithelial and mesenchymal phenotypes (28). EMT is physiologically required in different steps of embryogenesis, tissue morphogenesis during fetal development and wound healing (29). Furthermore, the activation of the EMT program in carcinoma cells is responsible for cell detachment from the primary tumor and acquisition of their invasive and migratory ability to distant sites.

EMT consists of a series of events orchestrated by a pool of transcription factors (EMT-TFs) including Zeb family members, Snail, Slug and Twist 1. These transcription factors induce downregulation of epithelial markers such as E-cadherin, occludins and cytocheratins while cause upregulation of mesenchymal markers such as vimentin, N-cadherin and fibronectin (30). These changes in gene expression will lead to loss of cell polarity, disassembly of cell-cell junctions, disruption of basal membrane and acquisition of enhanced migratory and invasive properties.

A number of molecules can activate the EMT program in cancer cells through the specific binding to membrane receptors that triggers signals to the nucleus with the consequent upregulation of EMT-TFs (**Figure 1B**). The signaling pathways that are known to activate EMT include WNT, Hedgehog, NOTCH, TGFβ, tyrosine kinase receptors and cytokine receptors downstream pathways (31). A cross-talk and cooperation between these pathways is also possible. Furthermore, stromal components of tumor microenvironment can secrete a variety of cytokines, chemokines and growth factors that can activate specific signaling pathways and induce EMT in cancer cells. Specific ligands such as TGFβ, IL6, VEGF, TNF can be released by stromal cells and promote the acquisition of a mesenchymal state by cancer cells.

NETs have been tested as EMT inducers in different normal and malignant cancer cells (**Table 1**). Pieterse et al. (32) showed that the exposure of microvascular and macrovascular endothelial cells to NETs caused a rapid loss of VE-cadherin followed by disassembly of cell-cell junctions and release of junctional β catenin. These events are elastase-dependent since they are inhibited by selective elastase inhibitors and are associated with the gradual development of a mesenchymal-like state in endothelial cells. Furthermore, the same authors showed that β catenin was translocated to the nucleus and caused upregulation of Snail1, one of the EMT-TFs. These observations were confirmed in a murine model of lupus nephritis and in kidney biopsies of patients with lupus nephritis concluding that NETs are major pathogenetic factors of this disease causing EMT in glomerular endothelial cells and consequent proteinuria, due to the loss of cell-cell junctions.

**Table 1:** Main experimental evidences and mechanisms of NET-induced EMT in different biological systems.

Experimental system	Observations	Signaling Pathways	Inhibitors	Ref.
Human umbilical vein endothelial cells (HUVECs) and conditionally immortalized glomerular endothelial cells (ciGEnCs)	• Loss of cell-cell contacts and impaired endothelial monolayer integrity • Acquisition of a spindle-shaped mesenchymal -like phenotype • ↓ VE- cadherin • ↓ CD31	Nuclear translocation of junctional β - catenin	Neutrophil elastase inhibitor	(32)
Breast cancer cells MCF-7	• More elongated fibroblast-like shape • Reduction of cell adhesion, and enhanced migratory properties • ↓ E- cadherin • ↑ N-cadherin, fibronectin, β - catenin • ↓CD24 and ↑CD44	ND	None	(33)
Non-small cell lung cancer cells A549	• ↓ E- cadherin • ↑ αSMA • ↑TGF-β, IL-1β and IL-8 in the presence of alveolar macrophages	TGF-β	None	(34)
Gastric cancer cells AGS	• Enhanced migratory properties • ↓ E- cadherin • ↑ Vimentin	ND	DNase I, PAD4 inhibitor	(35)
Colo-rectal cancer cells DLD1 and SW480	• Formation of pseudopodia • Alterations of the actin cytoskeleton • Enhanced migratory properties • ↓ E- cadherin, EPCAM • ↑ Vimentin, FN1, Zeb1, Slug	ND	None	(36)
Gastric cancer cells MKN-45 and MGC-803	• Enhanced migration and invasion • ↓ E- cadherin • ↑ N- cadherin, p-smad2	TGF-β	TGF-β inhibitor, PAD4 inhibitor, Neutrophil elastase inhibitor, DNase I	(37)
Non-small cell lung cancer cells A549 and SK-MES-1	• ↓ E- cadherin, lncRNA MIR503HG • ↑ Vimentin, N- cadherin • ↑ NLRP3, caspase 1, IL-1b, IL-18 • Increased phosphorylation of NF-kB	NF-kB	DNase I, MIR503HG	(38)
EGFR-driven non-small lung cancer cells (HCC827 and H1975) and breast cancer cells (MCF-7)	• Reduction of cell adhesion and enhanced migratory properties • Loss of epithelial phenotype • ↓ E- cadherin • ↑ Vimentin, Zeb 1, Slug, Snail, Twist 1 • ↑Notch 1 and cleaved-Notch 1	NOTCH 1	DNase I	(39)
Pancreatic ductal adenocarcinoma cells MIA Pa-Ca-2 and KPCY	• Increase of pseudopodia-like protrusions • Enhanced migration and invasion • ↑Snai 1, Snai 2 and Zeb 1 • Increased GTP-CDC42 and GTP-RAC1	ITGB1-ILK axis	ILK inhibitor and knockdown, DNase I, ITGB1 blocking Ab, CCDC25 silencing	(40)

↓, decrease; ↑, increase.

In agreement with these findings, Martins-Cardoso et al. reported that incubation of MCF-7 breast cancer cells with NETs caused drastic morphological changes, reduction of cell adhesion and enhancement of cell migration (33). Quantitative RT-PCR showed an enhanced transcription of Snai 1, Snai 2 and Zeb 1 genes in response to NETs exposure while immunocytochemistry analysis revealed a reduction of E-cadherin levels and an increased expression of fibronectin and β catenin. The same authors investigated the ability of NETs to induce changes in the expression of stem cell markers in MCF-7 cells and found that CD24 levels were decreased while CD44 expression was enhanced by NET exposure. Furthermore, the expression of a panel of proinflammatory cytokines including IL-1β, IL6 and IL-8 was enhanced in MCF-7 treated with NETs. Finally, using TGCA database, the authors found a positive correlation between the neutrophil-related signature gene expression with Snai 1 and β catenin genes.

In another study, Pandolfi et al. investigated the ability of NETs to induce EMT in lung epithelial cells (34). Based on the observations that bronchoalveolar lavage of severe COVID-19 patients contains high levels of NETs and that lung biopsies showed the expression of mesenchymal and epithelial markers in subgroups of pneumocytes, the authors co-culture A549 lung cancer cells with PMA-activated neutrophils or isolated NETs. After 24 h, they found upregulation of αSMA, a mesenchymal marker, and downregulation of E-cadherin. The same authors developed an *in vitro* airway model using the same cancer cell line co-cultured with human neutrophils, alveolar macrophages and infected with SARS-CoV2. They concluded that the activation of EMT program in lung cancer cells required SARS-CoV2 infection and the presence of both neutrophils and alveolar macrophages. These observations may have important implications for the pathogenesis of complications after SARS-CoV2 infection but also may shed light on NET-dependent induction of EMT in lung cancer cells.

Zhu et al. reported that exposure of gastric cancer cells to NETs caused downregulation of E-cadherin and upregulation of vimentin (35) along with an enhanced migratory ability. These effects were inhibited by DNase I and PAD4 inhibitor. Furthermore, the same authors developed a post−surgical residual tumor xenograft models and showed that xenografts of untreated mice contained NETs while animals treated with DNase I and PAD4 inhibitor did not show detectable levels of NETs. Furthermore, tumors of untreated mice showed the expression of vimentin and reduction of E-cadherin levels.

Further evidences came from Stehr et al. who examined the presence and intratumoral distribution of NETs in 85 samples of human colon cancer (36). They found that NETs were present in higher grade tumors often with loco-regional metastases and with increased local invasion. They also reported that DLD1 and SW480 colon cancer cell lines incubated with NETs showed cell morphology changes and alterations of the actin cytoskeleton. In addition, SW480 cells showed upregulation of vimentin, fibronectin, Zeb 1 and Slug whereas levels of E-cadherin and epithelial cell adhesion molecule (EPCAM) were decreased. The exposure of DLD1 cells to NETs enhanced the expression of fibronectin and Zeb 1. The induction of EMT in DLD1 cells was associated with an increased migratory capacity in wound healing assay.

Other authors addressed the issue whether postoperative abdominal infectious complications (AIC) can promote recurrence or metastases after radical gastrectomy for cancer through the release of NETs both in the peripheral blood and in the abdominal cavity (37). They found higher levels of NETs in serum, plasma and ascites fluid from patients with AIC than in patients without AIC. Furthermore, MKN-45 and MGC-803 gastric cancer cells co-cultured with neutrophils from AIC group showed enhanced proliferation, migration and invasion compared to those co-cultured with neutrophils from control or non AIC group both *in vitro* and in animal models. Also, a lower level of epithelial marker E-cadherin and higher level of mesenchymal marker N-cadherin were found in MKN-45 and MGC-803 gastric cancer cells when co-cultured with neutrophils from AIC group as compared to those co-cultured with control or non-AIC group. These observations were confirmed in animal models and in liver and peritoneal metastases of gastric cancer patients. The main driver of these effects was found to be TGFβ since NETs mediated E-cadherin downregulation, N-cadherin and p-Smad2 (nuclear) upregulation were abrogated by treatment with a TGFβ inhibitor such as LY2157299.

Wang et al. investigated the ability of NETs derived from non-small lung cancer (NSCLC) patients to induce EMT and the role of long non-coding RNA MIR503HG in modulating such process (38). They found that A549 and SK-MES-1 cells when treated with NETs expressed lower levels of E-cadherin and higher levels of N-cadherin and vimentin. In addition, their migratory and invasive abilities were enhanced. To test whether some long non-coding RNAs (lncRNAs) were involved in EMT induction, they performed a trascriptome RNA microarray analysis of A549 cells treated with NETs. They found a large panel of lncRNAs that were upregulated or downregulated upon cell exposure to NETs. In particular, the expression of lncRNA MIR503HG was dramatically reduced by 12 h treatment with NETs. Therefore, authors focused their attention on the overexpression or silencing lncRNA MIR503HG in NSCLC cells and found that this lncRNA inhibited the activation of EMT by NETs and reduced the formation of lung metastases in animal models. By analysing the differentially expressed genes induced by NETs, the authors found that several genes associated with inflammation were upregulated by NETs and these included NLRP3, Caspase1, IL-1b, and IL-18. Since NLRP3 and pro-IL-1 protein expression is upregulated by activation of NF-kB pathway, they tested whether NETs can activate the NF-kB pathway in A549 and SK-MES-1 cells and found increased levels of p50, phospho-p50, p65 and phospho-p65 after NET treatment. The activation of NF-kB pathway was inhibited by overexpression of lncRNA MIR503HG.

In a recent study (39), our group investigated the role of NETs as an adhesion substrate for a panel of cancer cell lines and the ability of NETs to induce EMT in the same cell lines. This study showed that cell adhesion to NETs was enhanced in all cancer cell lines and was mediated by the binding of α5β1 integrin and CCDC25 receptor expressed on the plasma membranes of cancer cells to fibronectin and DNA, respectively, included in the structure of NETs. Furthermore, the prolonged exposure of oncogene-driven lung cancer cell lines to NETs caused the activation of EMT program with reduction of E-cadherin expression and increased levels of vimentin, Zeb 1, Slug, Snail and Twist 1. In the same cell lines, a significant enhancement of cell migration was also observed. Then, in the effort to identify the signaling pathway involved in the activation of EMT, levels of Notch 1 and cleaved Notch 1 were tested in untreated and NET-treated lung cancer cells and increased levels of both molecules were found, indicating the central role of Notch 1 in NET-dependent activation of EMT especially in oncogene-driven lung cancer cell lines.

A more recent study reported the identification of NET-related gene expression signatures in human samples of pancreatic ductal carcinomas (40). In particular, the overexpression of NET-related genes containing several genes involved in integrin-actin cytoskeleton organization and activation of epithelial mesenchymal transition identified a subgroup of patients with very poor prognosis. Furthermore, exposure of pancreatic ductal adenocarcinoma cells (MIA Pa-Ca-2 and KCPY) to NETs enhanced cell migration and invasion and these effects were inhibited by DNase I, shRNA-mediated suppression of CCDC25 expression, antibodies targeting ITGB1 and ILK inhibitors. Exposure to NETs also resulted in a dose-dependent increase of EMT transcription factors including Snai 1, Snai 2 and Zeb 1 that was abrogated by silencing ILK. These findings highlighted the role of CCDC25-ITGB1-ILK signaling in NET-dependent activation of EMT program.

## Conclusions

A growing body of evidences indicates that NETs can induce EMT both in normal and malignant cells. This process is dependent from NET-associated neutrophil elastase and integrity of NET’s DNA backbone since neutrophil elastase inhibitors and degradation of DNA with DNase I prevent or reduce the occurrence of EMT in NET-treated cells. In cancer cells, NET-dependent activation of EMT program is associated with their enhanced ability to migrate, invade and disseminate at distant sites. Not all cancer cells when exposed to NETs showed activation of EMT program and this may depend on the differential sensitivity of cancer cells to the action of NETs that in turn may depend on the permissive status of their signaling network.

**Figure 2:**
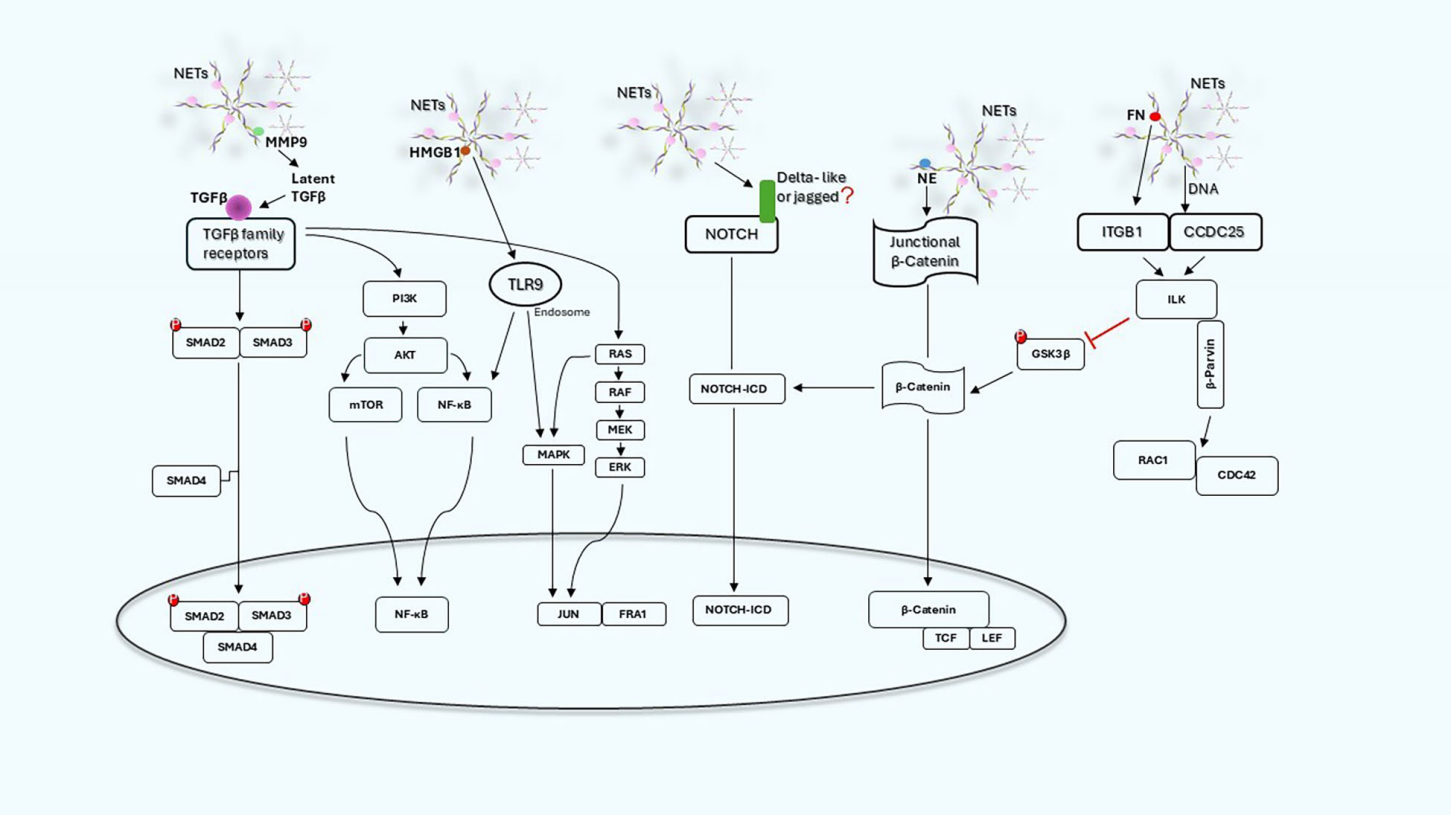
NET-induced signaling pathways and related cross-talk in cancer cells. Schematic graph illustrating the main signaling pathways engaged by NETs and how they can act independently or synergistically.

The signaling pathways involved in the activation of EMT in cancer cells exposed to NETs are not completely elucidated but, as reported above, several evidences indicate the involvement of β catenin, certain cytokines, TGFβ, NF-kB, Notch 1 and ITGB1-ILK axis that may act independently or synergistically (**Figure 2**). The involvement of multiple pathways and mediators is not surprising since EMT can be activated by different ligands and receptor pathways. What is surprising is that NETs contain in their complex structure a number of molecules that can trigger directly or indirectly EMT.

The composition of NETs can vary depending on several factors including the stimulus inducing NET formation, the disease status and even changes of physiological conditions of neutrophil donor (41, 42). Although the main structural components of NETs remain the same, the presence and the relative abundance of certain proteins can vary in different conditions. For instance, NETs generated by different stimuli through NADPH oxidase–dependent and -independent pathways may have different composition (42) and may trigger EMT with different intensity or through different signaling pathways. A comparative proteomic analysis showed divergences in protein composition of LPS-induced and spontaneously released NETs whereas PMA- and A23187-induced NETs showed a similar composition (43). Therefore, the heterogeneity in composition of NETs may explain their different ability to induce EMT in different studies and also the variability in the effectiveness of protease inhibitors in blocking NET functions.

In addition to the well-known protein markers of NETs such as citrullinated histones, neutrophil elastase and MPO, studies using proteomic analysis allowed to identify a large panel of proteins inside the structure of NETs. Urban et al. (44) reported a list of 24 proteins that have a nuclear, granular or cytoplasmatic localization in unstimulated neutrophils. In this study the authors focused on the functional activity of calprotectin as an inflammatory marker and anti-fungal NET-bound protein. More recent proteomic studies identified up to 2364 proteins (45) among which the most abundant were MPO, calcium and zinc-binding proteins, calprotectin and a group of RNA-binding proteins. Based on these studies reporting the enrichment of different groups of proteins belonging to different cellular compartments of activated neutrophils, it is likely that additional pathways may be found to be involved directly or indirectly in the activation of EMT program by NETs.

In addition to tumor associated neutrophils, other tumor microenvironmental components contribute synergistically to the activation of EMT program in cancer cells. The role of cancer associated fibroblasts (CAFs) has been well characterized and relies upon the secretion of high levels of various growth factors, cytokines and enzymes including TGFβ, matrix metalloproteinases, hepatocyte growth factor and urokinase-type plasminogen activator especially at the invasive front of tumors (46, 47). Similarly, activated tumor associated macrophages (TAMs) are able to induce EMT through the secretion of TGFβ and chemokine CCL18 (48, 49). The synergistic effect of these stromal cells is multiplied by the fact that some of the secreted factors may induce release of NETs from tumor associated neutrophils thus realizing a positive loop of interactions.

The studies reported here provide solid evidences that NETs can activate the EMT program in both normal and malignant cells. However, several aspects remains to be elucidated. For instance, it is unclear whether normal and malignant cells are equally responsive to NETs and whether the signaling pathways involved are the same. Similarly, not all epithelial cancer cell lines are equally sensitive to NET-dependent induction of EMT and despite the reported involvement of several signaling pathways, the interconnections among different signaling networks were not fully elucidated. Furthermore, the role of NETs in cancer should be evaluated taking into account the function of other components of tumor microenvironment such as cancer-associated fibroblasts, tumor associated macrophages and immune cells thus providing an integrated picture of the events occurring in tumor microenvironment. This integrated vision can contribute to identify the optimal therapeutic strategy to counteract NETs’ function.

NETs by inducing a mesenchymal state in cancer cells can determine their destiny. As drivers of EMT, NETs enhance the migratory and invasive properties of cancer cells promoting their dissemination at distant sites. Although incomplete, the mesenchymal state increases the heterogeneity of tumors and enhances their resistance to conventional anticancer therapies (47). In addition to the direct modulation of immune cells by NETs in tumor stroma (50), the acquisition of a mesenchymal state by tumor cells is associated with immune evasion and suppression through diverse mechanisms (51). In particular overexpression of Zeb 1 in response to an EMT inducer suppresses miR-200 expression that in turn causes upregulation of PD-L1 on cancer cells and T cell exhaustion (52, 53). Furthermore, the mesenchymal state is associated to upregulation and secretion of growth factors and other ligands that support the recruitment of immunosuppressive cells such as MDSC in tumor stroma (54).

Induction of EMT by NETs seems to occur through highly complex and redundant mechanisms thus posing a difficult challenge to the development of treatment strategies targeting NET-dependent EMT. Prevention of NET formation using PAD4 or neutrophil elastase inhibitors may deserve attention for future clinical applications. Alternatively, the disruption of NET structure by DNase I may be explored as treatment strategy in combination with standard anti-cancer therapies. Furthermore, the development of more selective targeted therapies may require the knowledge of specific signaling pathways activated by NETs in individual tumors.

In conclusion, NETs seem to have a leading role at the crossroad between inflammation and cancer with many important clinical implications and unraveling the complex network of their interactions with cancer, stromal and immune cells can provide suitable targets for preventing metastatic dissemination and increasing tumor response to chemotherapy and immune therapy.
